# The Impact of e-Health Literacy on Risk Perception Among University Students †

**DOI:** 10.3390/healthcare13030265

**Published:** 2025-01-29

**Authors:** Sonia Chien-I Chen, Menglu Yu, Yeqing Yu, Ruofei Wang, Zhaofei Zhu, Shuyan Liu, Guocong Zhang, Chung-Ming Own

**Affiliations:** 1 School of Economics, Qingdao University, Qingdao 266071, China; 2 College of Intelligence and Computing, Tianjin University, Tianjin 300072, China

**Keywords:** e-health literacy, public health crises, influencing factors, COVID-19 impact, digital health education, risk awareness

## Abstract

Background: The COVID-19 pandemic significantly increased public interest in e-health literacy, especially among university students. However, gaps remain in their ability to find and use credible online health information. Purpose: This study explores the effects of public health emergencies on e-health literacy among Chinese university students, aiming to identify influencing factors and propose solutions to improve digital health education. Methods: A structured survey using the eHEALS scale and additional questionnaires was administered to 300 students in Northern China. Statistical analysis was conducted using SPSS 25.0, focusing on literacy levels and their determinants. Results: The pandemic heightened interest in e-health, leading to modest improvements in literacy levels. However, challenges persist, including evaluating the credibility of information and addressing privacy concerns. Apps emerged as the most widely used e-health tools. Discussion: Findings highlight the importance of targeted health education programs to bridge gaps in e-health literacy and support students in effectively using digital health tools. The results emphasize integrating privacy safeguards and enhancing user trust in e-health systems. Conclusions: Enhancing e-health literacy can empower students to make informed health decisions, fostering better self-management and resilience during public health crises.

## 1. Introduction

The rapid development of digital technology has reshaped access to health information, making e-health literacy an essential skill in modern healthcare systems [1,2]. e-health literacy is the capacity to search for, understand, and evaluate electronic health (e-health) information to support informed health decisions [3,4]. Particularly in view of current public health crises, the relevance has greatly increased and emphasizes the need for people to effectively use online health platforms to support public health initiatives and personal health management [5].

Despite its growing importance, studies reveal significant disparities in e-health literacy across different demographic groups, particularly among young adults in non-Western contexts such as China [6,7]. Existing research highlights that university students, though technologically proficient, often struggle to critically evaluate and effectively use online health information [8,9,10]. Factors that enhance e-health literacy encompass studying health sciences, employing reputable professional websites, and allocating additional time to internet health information research [8,9]. Moreover, elevated literacy levels correlate with healthy lifestyle practices, including consistent physical activity. This study addresses these gaps by focusing on the impact of public health emergencies, such as the COVID-19 pandemic, on e-health literacy among Chinese university students.

Unlike prior studies that focus broadly on technological literacy, our research uniquely investigates the intersection of e-health literacy, behavioral patterns, and the sociocultural influences specific to Chinese university students. By employing the eHEALS scale and analyzing data across four distinct COVID-19 phases (2019–2023), we provide a granular understanding of how public health crises shape digital health engagement and literacy levels.

Yet, new data show a more complicated story [10]. Many university students underuse e-health tools, despite their technological expertise; they usually choose social networking and entertainment platforms over health-related applications. Against the conventional wisdom of e-health literacy as generally beneficial, some research has found a negative association between e-health literacy and individual innovation among health-related students [7]. These results draw attention to the trade-off between innovative problem-solving and information access. Interventions must thus be customized to improve e-health literacy while preserving creative ability.

With one of the biggest student populations in the world, Chinese students are using digitalization to transform the healthcare sector in a way influenced by their varied social, cultural, and educational backgrounds [11,12,13]. Though e-health literacy is becoming more and more important worldwide, very little is known about how public health crises, technological adoption, and cultural variables affect students’ interaction with digital health in China.

This study adopts the eHealth Literacy Model (eHEALS), developed by Norman and Skinner [14], as the theoretical framework. The model identifies six types of literacy (traditional, health, information, scientific, media, and computer literacy) essential for understanding and utilizing e-health tools effectively. This framework is particularly relevant in the context of the COVID-19 pandemic, where access to digital health resources became critical for health management.

The objective of this study is to examine the factors influencing e-health literacy among university students, focusing on their access to e-health resources, frequency of usage, and demographic characteristics during the COVID-19 pandemic. The main goal of this study is to find the causes of Northern Chinese college students’ curiosity in e-health during the COVID-19 epidemic. This study clarifies the interaction among college students’ e-health literacy, behavioral patterns, and bigger educational settings, so augmenting what is already known. The declared objectives of the report are to help young people to properly self-manage their health, raise their e-health literacy, and inspire innovation.

## 2. Methods

### 2.1. Study Design

This cross-sectional study was conducted to explore factors influencing Northern Chinese university students’ e-health literacy during the COVID-19 pandemic. Data were collected via online questionnaires administered between 2019 and 2023 and correspond to key phases of the COVID-19 pandemic, including the outbreak, containment measures, variant surges, and policy liberalization. These phases provide a comprehensive framework for analyzing changes in e-health literacy over time.

This study utilized a stratified random sampling method to select 300 university students aged 18–25 years from Northern China. This approach was employed to ensure proportional representation across key demographic and academic subgroups, such as gender and academic discipline, capturing a diverse and representative sample of the university student population. By stratifying the sample based on these characteristics, the method effectively minimized selection bias and enhanced the generalizability of the findings to a broader population.

The participants were drawn from universities in Shandong Province, chosen for its demographic diversity, encompassing both urban and rural populations, and its role as a prominent educational hub in Northern China. These features provided an inclusive context for examining e-health literacy among students from varied backgrounds.

The sample size of 300 participants was determined based on a review of prior studies on e-health literacy, ensuring adequate statistical power to derive meaningful insights. The stratified sampling approach further ensured balanced representation across academic disciplines and gender groups. These methodological choices align with this study’s objectives, contributing to a robust and representative research design.

Data were gathered by looking at search frequency and content using the keyword “e-health”, particularly with an eye on university students as users to assess e-health involvement. The period commenced with the COVID-19 pandemic in 2019, during which public interest in digital health technologies surged significantly. Three hundred online questionnaires were administered to university students in Shandong province. The poll gathered information on health status, educational background, academic qualifications, age, gender, perceived health, income, region, and professional field.

This strategy is employed as it can assist to categorize temporal and geographical characteristics of how pandemic circumstances influenced students’ e-health knowledge and involvement. The high-quality education institutions and diverse student populations in the Northern China made these processes perfect for study. The findings seek to inform worldwide conversations on improving e-health awareness and participation.

To ensure participant anonymity and data security, several measures were implemented. Participants’ responses were anonymized by assigning unique identifiers, with no personally identifiable information (PII) collected during the survey. The data were encrypted and stored on password-protected systems accessible only to the research team. The survey was conducted anonymously online, and participants were informed during the consent process that their responses would remain confidential and be used solely for research purposes.

### 2.2. Participants and Investigation Method

The study population consisted of 300 university students in Northern China. The participants’ ages ranged from 18 to 25 years, with a mean age of 21.4 years (Standard Deviation = 1.6). Regarding their educational background, 91.57% were undergraduate students, 4.82% were specialists, 2.81% were postgraduate students, and 0.8% were doctoral candidates.

The survey comprised three components: the “Electronic Health Literacy Survey Scale (eHEALS)”, the “Basic Information Survey Form”, and the “COVID-19 Question-naire on the Influencing Factors of Sub-health Literacy” [14,15,16]. Trained staff members supervised questionnaire completion to guarantee uniformity and correctness. Each participant received normal instructions and the survey was taken anonymously. Participants were reassured that their answers would remain private and the aim and relevance of this study were explained before we started. A total of 249 genuine replies were gathered for study out of the sent-out questionnaires.

To ensure data validity and reliability, responses were reviewed based on specific exclusion criteria. First, responses containing logical inconsistencies, such as contradictory answers to related survey items (e.g., selecting both “Strongly Agree” and “Strongly Disagree” for items assessing ease of e-health tool use), were excluded. Second, incomplete responses were removed if more than 20% of the survey items were left unanswered. For example, participants who did not respond to six or more items on the eHEALS scale were excluded. Third, individuals who failed to meet inclusion criteria, such as those outside the target age range of 18–25 years or those who were not university students, were excluded. These criteria ensured that the dataset was both robust and representative of the target population.

The eHEALS instrument utilized in this study comprises eight items measured on a 5-point Likert scale, ranging from 1 (“Strongly Disagree”) to 5 (“Strongly Agree”). This scale was chosen to balance simplicity and the ability to capture meaningful variability in responses. While broader scales (e.g., 7- or 10-point) might offer finer distinctions, they could increase cognitive load, potentially affecting reliability in a student population. Conversely, narrower scales (e.g., 3-point) might oversimplify responses, reducing the sensitivity of the measure. The 5-point scale has been validated in prior studies using the eHEALS, ensuring comparability and interpretability of findings across contexts.

The use of small incentives, such as gift cards or vouchers, was carefully considered to encourage participation while minimizing the risk of response bias. To ensure that the incentives did not unduly influence participants’ responses, several measures were implemented. First, participants were informed that the incentives would be provided unconditionally, regardless of their answers or level of participation, thereby reducing any potential pressure to provide responses aligned with perceived expectations. Additionally, the survey was conducted anonymously, and no identifying information was collected or linked to the responses. This anonymity ensured that participants felt secure in providing honest and unbiased answers. Furthermore, clear instructions were included at the beginning of the survey, emphasizing the importance of responding truthfully and based on their genuine experiences and perceptions.

While these measures helped to mitigate potential bias, we acknowledge that the use of incentives could still influence this study in subtle ways, such as by increasing participation among specific groups more motivated by such rewards. This consideration has been addressed in the revised manuscript, where we have discussed the potential implications of incentives on the results and outlined the steps taken to minimize any associated bias. We hope this clarification addresses the reviewer’s concerns and strengthens this study’s methodological transparency.

### 2.3. Data Analysis

This study uses eHEALS to assess individuals’ ability to find, understand, evaluate, and use online health information effectively. The scale comprises eight items divided into three dimensions: First one is the application ability: It is the dimension to measure the capacity to find, obtain, understand, and utilize online health information and services. The second ability, assessment ability, measures an individual’s capability to discern and evaluate the quality of online health information. The third capacity is decision-making ability, utilized to evaluate the capability to make informed health decisions based on internet information. We use a 5-point Likert scale for each item, and the possible total scores are 8–40. If your score is more than 32, that means you may have a comprehensive understanding of e-health.

In this study, we looked at e-health literacy in four different time periods: (1) the 2019 COVID-19 epidemic, (2) the 2020–2021, effective containment of the virus, (3) the 2022 revival of mutant variations, and (4) the 2023 liberalization of COVID-19 policy. Temporal comparisons using the scale highlighted changes in university students’ interest in e-health and their literacy levels during these distinct phases. The scale has demonstrated strong reliability and validity, having been widely applied among students, patients, and older adults in China.

Health, education, age, sex, income, geography, and occupation were studied in this study. A unique twenty-three-item questionnaire examined emotional, instrumental, informational, and social member support. On the 5-point Likert scale, 1 denotes complete disagreement and 5 represents entire agreement, higher scores indicate stronger social support. An application was put in place to continuously turn the pages to keep data collecting running smoothly.

In order to encourage more involvement, a few incentives were given out. Each respondent was asked to complete all questions, and each participant was limited to one response, in order to avoid data duplication. The evaluation of replies during data collection was performed using both internal and external criteria. We did not include entries that included logical fallacies or did not meet our inclusion requirements.

For the sake of precision, data were entered using Epi-Data 3.1 and analyzed using SPSS 25.0. The model’s approach relied on the least squares methodology. Factors such as gender, place of origin, frequency of use of e-health products after COVID-19, effects of the pandemic, and challenges with self-management of e-health were considered independent factors. The ability to describe electronic health was the one being studied. Using linear regression, we were able to ascertain the nature of the relationship between these variables.

A linear link was suspected by analyzing the F-values to see if the regression coefficient substantially differed from zero. Model fit was assessed using R^2^ and VIF values. Ridge regression or stepwise regression were advised for collinearity (VIF > 10 or strictly > 5). Variables with a significance level of *p* < 0.05 were further analyzed to assess their impact on the dependent variable. By comparing regression coefficients, the degree of influence of each independent variable was determined, which enabled the formulation of an appropriate model.

## 3. Results

This section presents the findings organized into key themes reflecting the primary dimensions of this study. These themes—(1) behavioral factors influencing e-health literacy, (2) the role of resource availability in literacy development, and, and (3) demographic Influences on e-health literacy—were selected based on this study’s objectives to comprehensively address the research questions. This thematic organization allows for a structured analysis, emphasizing the interplay between literacy, behavior, and external factors. The scale demonstrated high reliability in this study, with an overall Cronbach’s α coefficient of 0.91 and subscale coefficients ranging from 0.82 to 0.89. This well-validated instrument has been widely applied across diverse populations, including students, patients, and older adults, further supporting its reliability and validity [17,18].

### 3.1. Behavioral Factors Influencing e-Health Literacy

Regression analysis revealed that an increase in the frequency of e-health product usage post-COVID-19 outbreak was a significant predictor of e-health literacy (B = 0.214, *p* < 0.001). Participants who reported higher usage demonstrated significantly greater literacy levels. Reliability analysis further indicated strong internal consistency across behavioral items, with a Cronbach’s alpha of 0.92.

### 3.2. Resource Availability and e-Health Literacy

**Figure 1:**
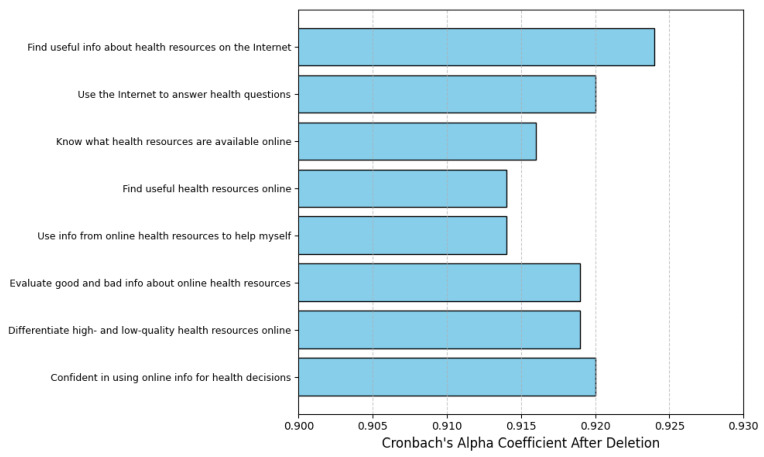
Cronbach’s Alpha Coefficients for e-Health Literacy Items.

Access to e-health resources was positively associated with higher literacy levels (B = 0.023, *p* < 0.001). Participants with consistent access to digital tools and reliable internet reported a stronger ability to evaluate and utilize health information online. Figure 1 illustrates the distribution of literacy scores by resource availability, highlighting the disparity between those with and without access.

### 3.3. Demographic Influences on e-Health Literacy

Demographic variables such as gender and place of origin had minimal impact on e-health literacy. While gender showed a small positive association (B = 0.09, *p* < 0.05), academic qualifications and place of origin were not significant predictors (*p* > 0.1). This sub-section provides a detailed breakdown of demographic influences and corresponding statistical significance levels.

#### 3.3.1. Exercises on Reliability and Analytical Steps

The validity and consistency of the questionnaire scales were evaluated by means of a reliability study. The questionnaire’s contents were considered as independent variables; Cronbach’s alpha was used as the consistency and dependability evaluation tool. This approach guarantees that the obtained measurements for the research produce trustworthy results.

**Figure 2:**
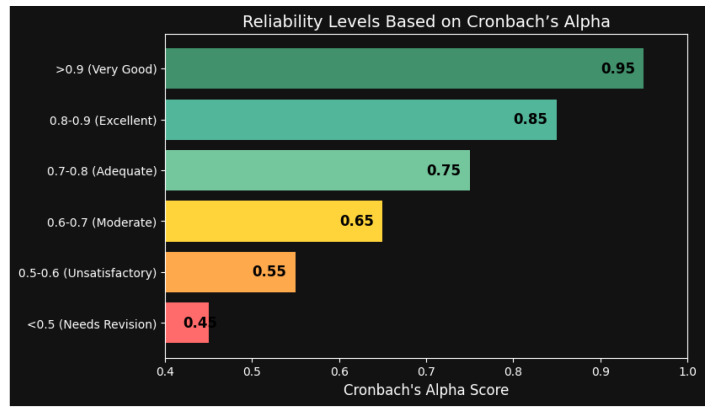
Reliability Levels of Questionnaire Based on Cronbach’s Alpha.

Despite the lack of a consensus on how to interpret Cronbach’s alpha, most academics do agree on broad dependability levels. Figure 2 illustrates that a Cronbach’s alpha score above 0.9 indicates very good reliability, while a score between 0.8 and 0.9 signifies excellent reliability. Scores in the range of 0.7–0.8 suggest adequate dependability, whereas scores in the range of 0.6–0.7 indicate moderate reliability. Reliability is considered unsatisfactory when the score falls between 0.5 and 0.6. If the numbers are less than 0.5, the questionnaire has to be significantly revised.

To improve the scale, each item underwent further study to uncover factors that might potentially compromise its overall dependability. The item was deemed eligible for removal if the “corrected item-total correlation” fell below 0.3 or if the “alpha coefficient after item deletion” significantly exceeded the initial alpha coefficient. The technique enhanced the dependability of the questionnaire.

#### 3.3.2. Demographic Characteristics of Participants and Analysis Results

**Figure 3:**
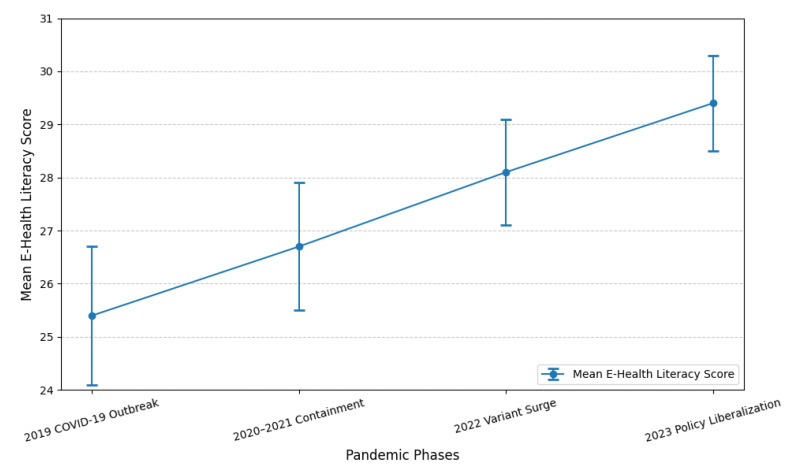
Trends in e-Health Literacy Scores Over Time.

The demographic characteristics of this study population reveal a clear predominance of young adults as shown in Figure 3. A majority of the participants were undergraduate students, accounting for 91.57% of the total sample. This highlights that this study predominantly targeted individuals pursuing their bachelor’s degree, representing a diverse mix of academic disciplines and backgrounds. In terms of age distribution, the majority of participants fell within the 18–22-year age range, comprising 78% of the sample. This age range aligns with the typical demographic of undergraduate university students in Northern China. The remaining participants included postgraduate students (2.81%), specialists (4.82%), and doctoral candidates (0.8%), indicating a smaller but diverse representation of advanced learners and professionals. This demographic composition ensures that the findings are reflective of the broader university student population, particularly younger adults actively engaged in higher education.

Cronbach’s alpha coefficients for the e-health literacy questionnaire are shown in Table 1, indicating that the instrument was dependable for this study. Overall, Cronbach’s alpha was 0.928, and summarized it was 0.931. This provides strong proof of internal consistency. A total of 249 individuals completed the eight-item survey. The results indicate that the survey is a dependable and effective instrument for assessing e-health literacy among university students. Subsequent research may be undertaken confidently with this scale, since its elevated Cronbach’s alpha indicates that the items reliably measure the target construct and are well aligned.

The statistical analysis of the removed items is summarized in Table 2. Alpha and general corrected item-total correlation (CITC), which measure the consistency of each item with the entire scale, both rose when the item “I know how to find useful information about health resources on the Internet” was removed. This implies that the question could did not fit well with the general scope.

Likewise, after removing the item “I know how to use the Internet to answer my health questions”, the general CITC and alpha coefficient increased, suggesting that scale adjustments could increase its dependability and accuracy. In order to ensure that the scale consistently measures e-health expertise, these results highlight the necessity to refine certain items.

The total CITC correlation and alpha coefficient increased after removing the item “I know where to find useful health resource information online”, indicating potential locations for scale adjustment. Removing the item “I am able to differentiate between high- and low-quality health resource information online” similarly showed improvements in the general CITC correlation and alpha coefficient; nevertheless, no more scale adjustments were judged required. Confirming no need for changes, the CITC correlation and alpha coefficient stayed high when the item “I am confident in using online information for health decisions” was eliminated.

Figure 1 depicts Cronbach’s alpha coefficients after deletion for each item on the e-health literacy scale. These coefficients provide insight into the internal consistency of the scale and how each item contributes to its overall reliability. The results demonstrate that all items have high Cronbach’s alpha values, exceeding 0.91 after deletion, which indicates excellent internal consistency throughout the scale.

Among the items, “I know how to find useful information about health resources on the Internet” stands out with the highest Cronbach’s alpha coefficient after deletion (0.924). This suggests that while the item is highly correlated with others in the scale, its removal slightly improves the overall reliability, highlighting potential redundancy in its contribution to the construct of e-health literacy. Similarly, items such as “I know how to use the Internet to answer my health questions” and “I am confident in using online information for health decisions” also exhibit strong Cronbach’s alpha values after deletion (0.92 each), indicating their robustness and consistency with the scale.

Other items, including “I know what health resource information is available from the Internet”, “I know where to find useful health resource information online”, and “I know how to use the information I get about online health resources to help myself”, display slightly lower alpha values (approximately 0.914). However, their consistently high reliability scores suggest they contribute meaningfully to the scale while maintaining low redundancy.

Overall, the high Cronbach’s alpha coefficients across all items reflect the scale’s exceptional internal consistency and reliability. The item “I know how to find useful information about health resources on the Internet” is particularly notable for its strong relationship with other items while slightly overlapping in the measurement of e-health literacy. These findings confirm the robustness of the scale in evaluating digital health competencies.

### 3.4. Consequence Analysis

#### 3.4.1. Single-Factor Analysis of e-Health Literacy Scores

The results show that, with an average item score of 4.00 ± 1.06, university students’ general e-health literacy score was 28.12 ± 8.46. Among the individual questions, “I know where to get useful health resource information online” got the highest average score of 3.59 ± 0.99 but “I know how to find useful health resource information online” had the lowest average score of 3.31 ± 1.20 as shown in Table 3. The findings indicate differences in students’ perceived competencies in various dimensions of e-health literacy.

The mean e-health literacy scores demonstrated a gradual increase across the four pandemic phases as shown in Figure 3. During the COVID-19 outbreak in 2019, the average score was 25.4 ± 1.3. This score rose to 26.7 ± 1.2 in the containment phase (2020–2021) and 28.1 ± 1.0 during the variant surge (2022), and then peaked at 29.4 ± 0.9 in the policy liberalization phase (2023). These trends suggest an increasing familiarity with digital health tools and information among the participants, likely influenced by the growing reliance on e-health services during the pandemic. Figure 3 provides a visual representation of this progression.

#### 3.4.2. Changes in the Use of e-Health Products Following the COVID-19 Outbreak

Following the COVID-19 outbreak, there was a notable increase in e-health usage among university students, with 60.64% indicating higher utilization. Nonetheless, e-health-related applications were already extensively utilized by students, with 30.92% indicating no change in usage frequency, as illustrated in Table 4. The findings underscore the substantial influence of the pandemic on the uptake of e-health tools, while also indicating a pre-existing familiarity with these technologies prior to the pandemic.

The “Classification of e-health product usage among participants” table summarizes the various e-health products used, together with the user counts and corresponding ratios. Apps were the most popular e-health tool among participants, with 163 users accounting for 29% of the total. Then, there were electronic bracelet watches, worn by 131 people, or 24% of all the participants. Popular mini programs like Meiyu, Ping An Good Doctor, and Lilac Doctor drew 130 participants totaling 23%. Finally, one hundred participants—eighteen percent of the total—used short video services like Douyin. Table 5 shows the different tastes for e-health solutions; among the participants, mobile applications are the most often used technology.

This study looked at the population as a whole to find out what factors were most important for improving e-health literacy. Table 6 shows that the most important components, comprising around 30% of the total, were risk area segmentation and national policy direction. These results highlight the need to bolster institutional support and capitalizing on government programs to encourage the efficient use of e-health technology.

We used the international e-health literacy scale to measure the degree to which college students understood and could use electronic health records. As indicated in Table 7, 41.37% of students showed a limited capacity to locate helpful information on health resources on the Internet when asked about this skill. But more than half of the class could not find or verify the accuracy of health-related websites. These results show that college students have a lot of work to do when it comes to understanding and using e-health services, thus there has to be an effort to help them out specifically.

The pharmaceutical industry’s platforms disseminate both accurate and misleading COVID-19 information, which makes it difficult to distinguish between credible sources. According to Table 8, over half of the respondents (46.99%) were able to differentiate between high- and low-quality health information, with 22.49% stating they were “relatively vague” in their understanding. “These results imply that most university students lack the ability to clearly separate accurate from deceptive health information available online.

This survey study examined the primary obstacles in e-health development as stated in Table 9 to increase e-health literacy among university students and match e-health goods with their needs. The findings imply that people give the security and privacy of personal health data high importance, and they obviously demand the development and improvement of innovative medical technology and services.

In this survey, 249 valid questionnaires were obtained, of which the male to female ratio was 7:13 and the urban to rural ratio was 1:1. Among all participants, 4.82%, 91.57%, 0.8%, and 2.81% were specialists, undergraduates, postgraduates, and doctoral students, respectively.

Table 10 summarizes the results of the linear regression analysis, which examines the factors influencing participants’ familiarity with the concept of e-health. Significant predictors identified include changes in the frequency of e-health product usage following the COVID-19 outbreak (B = 0.214, *p* < 0.001) and access to e-health resources (B = 0.023, *p* < 0.001), both demonstrating positive associations. The findings indicate that an increased frequency of e-health product usage post-outbreak was a significant determinant of higher e-health literacy levels (*p* < 0.001). Furthermore, access to e-health resources was positively and significantly associated with enhanced e-health literacy (*p* < 0.001), underscoring the importance of resource availability in fostering digital health competencies.

**Figure 4:**
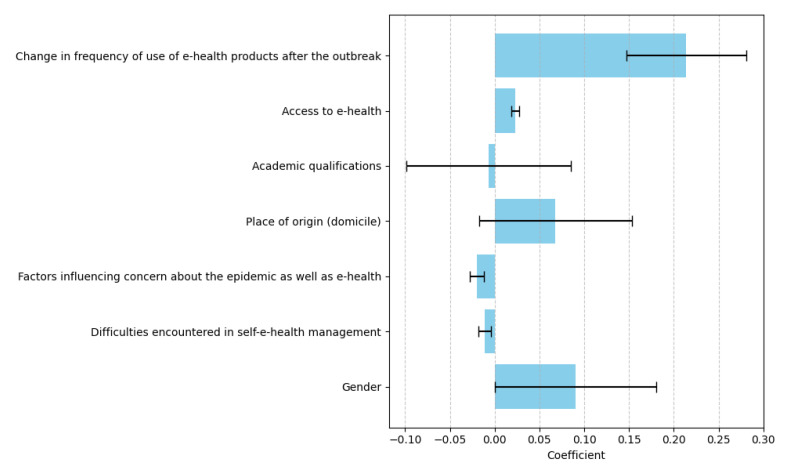
Regression Coefficients and Predictors of e-Health Literacy.

Figure 4 titled “Regression Coefficients and Predictors of e-Health Literacy” illustrates the results of a linear regression analysis, identifying the factors that influence participants’ familiarity with the concept of e-health. The coefficients for each predictor are presented alongside error bars that indicate their standard errors, providing a clear depiction of both the magnitude and reliability of these associations.

The most prominent finding is the strong positive relationship between the change in frequency of e-health product usage after the outbreak and e-health literacy. This factor, with a coefficient of B = 0.214 (*p* < 0.001), emerges as the most significant predictor. It suggests that participants who increased their usage of e-health products during the COVID-19 outbreak were more likely to exhibit higher levels of e-health literacy. Similarly, access to e-health resources demonstrates a significant positive association with e-health literacy, with a coefficient of B = 0.023 (*p* < 0.001). This finding underscores the crucial role of resource availability in enabling individuals to improve their familiarity and competence with digital health tools.

Other factors, such as gender, show a moderate positive influence on e-health literacy, with a coefficient of B = 0.09 (*p* < 0.1). Although its effect is smaller, gender appears to have a minor role in shaping participants’ understanding of e-health concepts. In contrast, factors related to concerns about the epidemic and e-health have a small negative coefficient of B = −0.02 (*p* = 0.017). This indicates that heightened concern may slightly hinder participants’ ability to engage with and comprehend e-health, although the impact is relatively modest.

Several predictors, including academic qualifications and place of origin, exhibit little to no significant effect on e-health literacy. Academic qualifications have a coefficient of B = −0.007 (*p* = 0.936), while place of origin (domicile) has a coefficient of B = 0.068 (*p* = 0.424). These findings suggest that demographic characteristics may not play a substantial role in influencing digital health competencies. Similarly, difficulties encountered in self-e-health management show a small negative association (B = −0.011, *p* = 0.086), indicating that self-management challenges might slightly lower e-health literacy, though the effect is not statistically significant.

Overall, the results highlight the critical importance of behavioral and resource-related factors, such as frequency of use and access to e-health tools, in predicting e-health literacy. Demographic factors, on the other hand, appear to have a limited role. These findings suggest that targeted strategies to improve resource accessibility and encourage regular engagement with e-health tools may be effective in enhancing digital health competencies. The figure visually reinforces these conclusions, providing a clear summary of the predictors and their respective impacts on e-health literacy.

Regarding factors such as concern about the epidemic and e-health (B = −0.02, *p* < 0.05) and difficulties in self-e-health management (B = −0.011, *p* < 0.1) showed negative associations with familiarity. As for gender factor, it had a notable but less significant impact (B = 0.17, *p* < 0.1). Variables such as academic qualifications and place of origin did not demonstrate significant effects on participants’ familiarity with e-health.

Overall, the adjusted R^2^ value of 0.168 and an F-value of 8.155 (*p* < 0.001) suggest the variance in participants’ familiarity with e-health. These results imply the need for access to e-health resources. Its frequency of usage also emphasizes its part in forming knowledge and points out areas for more intervention, including improving self-management techniques and handling issues connected to pandemics.

The F-test findings reveal a *p*-value of 0.000 ***, therefore disproving the initial theory that the regression coefficient is 0. Therefore, the model meets the relevant requirements. Regarding the performance of the co-linearity variable, VIF is less than 10, which indicates that the model is well constructed and has no problems with multiple co-linearity.

The equation of the model is as follows: y = 1.32 + 0.214 (change in frequency of using e-health products after the outbreak) + 0.023 (access to e-health) **. This is further adjusted by subtracting 0.007 * (education), 0.02 * (factors influencing concern about the outbreak and e-health), and 0.011 * (difficulties in self-e-health management). In addition, 0.068 * (place of birth) and 0.17 * (gender) are added to complete the model equation.

## 4. Discussion

### 4.1. Key Findings

#### 4.1.1. The Importance of e-Health Literacy

e-health literacy is essential for equipping individuals with the skills to effectively navigate digital health environments. This subsection addresses the low levels of e-health literacy observed among university students, highlighting their challenges in evaluating and applying online health information. These findings are consistent with existing literature, which identifies similar difficulties across diverse populations. This study demonstrates that enhancing university students’ e-health literacy could significantly improve their ability to recognize and respond to health-related risks.

The significant *p*-values (*p* < 0.001) observed for the frequency of e-health usage and access to resources underscore their pivotal role in improving e-health literacy. This supports previous research indicating that frequent engagement with digital health tools fosters familiarity and competence, as presented in Table 10. Additionally, the ability to conduct accurate risk assessments strongly correlates with the capacity to critically evaluate online health information.

These findings emphasize the critical importance of e-health literacy in empowering individuals to make informed medical decisions in increasingly digitalized healthcare contexts.

#### 4.1.2. The Influence of Public Health Crises on e-Health Engagement

Public health emergencies, such as the COVID-19 pandemic, have profoundly influenced individuals’ interactions with digital health tools. This subsection explores the increased adoption of e-health products during the pandemic, highlighting the role of crises in accelerating the uptake of digital health technologies. Despite this growth, persistent barriers to effective engagement remain, including concerns over data privacy and the quality of information available.

This study demonstrates a significant rise in Chinese university students’ interest in e-health tools during the COVID-19 pandemic. However, consistent with findings from other studies, there is a nuanced picture of how effectively students utilize their technical skills during public health emergencies. Notably, 60.64% of students reported increased usage of e-health products during the pandemic, underscoring the role of the crisis in fostering digital health adoption. Among these tools, mobile applications emerged as the most frequently used, accounting for 65.46% of e-health engagement, further emphasizing their central role in students’ interactions with digital health resources.

This analysis highlights both the opportunities and challenges in promoting e-health engagement during public health crises, offering insights for improving digital health literacy and overcoming existing barriers.

#### 4.1.3. Implications for Policy and Educational Interventions

The findings of this study have significant practical implications for developing targeted policies and educational programs aimed at improving e-health literacy. This subsection examines strategies for integrating e-health literacy training into academic curricula and public health campaigns while addressing systemic barriers, such as limited access to reliable digital infrastructure and low user trust in e-health technologies.

A key result revealed that more than half of university students struggled to obtain and recognize reliable online health information. Despite demonstrating general proficiency with computers, many students faced challenges in assessing the credibility of health resources, highlighting a substantial gap in e-health literacy. These findings underscore the importance of equipping students with the skills to critically evaluate and effectively utilize diverse online health tools. The average e-health literacy score of 28.12 ± 8.46, as measured in this study, indicates a relatively low level of proficiency. Notably, the item “I know where to get useful health resource information online” received the highest average score (3.59 ± 0.99), while “I know how to find useful health resource information online” had a lower average score (3.31 ± 1.20).

The use of a 5-point Likert scale in this study provided a practical balance between simplicity and granularity in measuring e-health literacy. While broader scales might offer finer distinctions, they could introduce additional complexity, particularly in a student population. Future studies could consider exploring broader or narrower scales to evaluate their impact on the precision and reliability of eHEALS responses.

These findings highlight the urgent need for targeted educational initiatives to improve e-health literacy. It is essential to teach students how to critically assess and effectively use health information found online. Additionally, addressing concerns about data privacy and security is critical for building user trust and encouraging greater adoption of e-health solutions. By fostering collaborative efforts between policy makers, educators, and healthcare providers, we can equip young adults with the necessary knowledge and resources to navigate the digital healthcare landscape confidently and make informed decisions regarding their health and well-being.

### 4.2. Comparison with Existing Research

The results are matched in this subsection with present research. Similarities and differences are emphasized to go over why variances could arise. This will enable the position of this work within the larger field of research.

#### 4.2.1. e-Health Literacy Among University Students

This paper fills in important knowledge gaps on e-health literacy in the framework of Chinese university students. Previous studies have not paid this group enough attention. Emphasizing this subgroup, this study clarifies the particular cultural, social, educational, and demographic elements influencing digital health involvement in China.

The findings of this study confirm those of other studies demonstrating that health literacy levels are much influenced by gender, age, educational level, and socioeconomic status. This study underlines the possibilities of education in promoting e-health literacy, especially when developing focused health literacy interventions [10,19], therefore addressing the several demands of students. Interdisciplinary resources may be used in the framework of university education to provide customized courses that not only improve general health literacy but also equip students to critically assess and implement digital health solutions efficiently.

While prior studies in Western populations reported a moderate association be-tween e-health literacy, and risk perception, university students often exhibit lower health literacy scores compared to reference populations, our findings suggest a stronger correlation among young adults in developing regions [10,20]. Health literacy initiatives should also be flexible enough to accommodate the needs of certain student populations, as this study confirms [21]. To address the growing demands of a digitalized healthcare system, these results support the idea that health education programs should incorporate e-health literacy into them.

#### 4.2.2. Impact of Public Health Crises on e-Health Engagement

The public health crises like COVID-19 pandemic highlighted the increasing relevance of e-health literacy, which Eysenbach, (2020) defined as a major instrument in ad-dressing the issues of disinformation during a health crisis [22]. Beyond its immediate health consequences, e-health literacy fits more general debates on the transforming power of technological innovation in tackling world issues like sustainable energy transitions and society advantages via developments [23].

Particularly among older persons, previous studies including Ghazi et al. [24] have investigated the relationship between e-health literacy and elements including perceived health status and psychological well-being. Building on this basis, the current research turns the emphasis to investigate how public health emergencies, including the COVID-19 epidemic, affect variations in e-health literacy [25,26,27,28]. This approach broadens our knowledge of e-health literacy, and it emphasizes its fluidity and ability to develop in the face of societal upheaval.

The findings also have ramifications for how breakthrough technology, such as blockchain, might be applied more extensively to address critical social challenges. With its capacity to reduce transaction costs, improve traceability, and ensure data quality, blockchain technology has proven to be a vital tool in e-health and carbon reduction programs alike [29]. With blockchain technology, healthcare organizations can ensure the security of patient information, facilitate the transfer of data easily, and keep digital health records intact [30,31]. These tools enable people to make more informed medical decisions, therefore proving how technology can be used to enhance e-health system results and openness.

Unlike past research mostly focused on demographic or technical factors, this analysis finds the epidemic as a major driver of higher digital health participation [4,10,20]. It emphasizes how individual capacity and desire to interact with e-health technologies are shaped by the particular circumstances of a public health emergency. From this vantage point, the dynamic interaction between digital health uptake and outside problems becomes clearer.

#### 4.2.3. The Effect of Cyberchondria Behavior on e-Health Literacy

This research advances understanding of e-health literacy in several contexts. Previous studies have indicated that e-health literacy helps students, late teens and healthcare professionals as well as global health systems. Özer et al. [32] looked at how e-health literacy among healthcare professionals was affected by cyberchondria. According to the study, employees who spend too much time searching for health information online may have trouble identifying reputable sources. As stated in [32], this highlights the need to implement tailored treatments with the objective of enhancing critical assessment skills. This is especially important for people who must manage large amounts of digital information, which aligns with our results on the issues encountered by healthcare providers.

Similarly, Masilamani et al. [33] reported that late teens in India rely on cellphones to get health information. The study indicated gaps in their capacity to assess the reliability and quality of health information, which is consistent with our results concerning the younger population’s difficulties with information quality in digital health contexts. This emphasizes the need to provide age-appropriate e-health literacy training.

According to Alsahafi et al. [34] e-health literacy significantly influenced users’ acceptance of electronic personal health records in Saudi Arabia. Their results indicate that e-health literacy could enhance the adoption of integrated health technologies. Our study echoes this, and it is suggested that e-health literacy is a critical enabler for leveraging e-health records, especially in resource-limited settings.

Building on the research by Zhao et al. [35], who identified sociodemographic factors as the most influential, this study presents a thorough analysis of the e-health literacy of Chinese internet users. Our findings are supported by their cross-sectional approach, which confirms the impact of internet access and educational achievement on e-health literacy.

Further, the research of e-health education was conducted in Saudi Arabia’s medical colleges, which reveals gaps in formal training for health informatics. This sup-ports our recommendation for integrating e-health literacy modules into medical education to prepare future professionals for a digitally transforming healthcare environment [36].

Okan et al. [37] conceptualized health literacy as a “social vaccine” during the COVID-19 pandemic by underscoring its preventive potential against misinformation and its role in promoting public health behaviors. This broader perspective enriches our findings by situating e-health literacy as a pivotal public health tool.

At last, Tran et al. [38] looked at mental health outcomes and e-health literacy among nursing students. Higher e-health literacy related to lower anxiety, despair, and preventative behavior. This is pertinent to the consequences of our results and supports initiatives meant to increase e-health literacy, therefore helping not only with regard to psychological well-being but also with relation to medical results.

Through investigating the temporal effects of COVID-19 on e-health literacy, this study provides a better knowledge of how behavioral changes during health emergencies support enhanced literacy and involvement. It underlines the transforming power of public health crises in determining how people engage with digital health services, thereby providing important information for next actions and policy development.

### 4.3. Implications

Not only is increasing health awareness of importance, but also e-health literacy usage affects other aspects. These findings suggest that if e-health literacy training were taught in schools, young people’s risk awareness and health outcomes may be much raised. Moreover, public health campaigns should focus on supporting the evaluation of credible internet health information to help to foster better risk management practices. This result thus has important consequences for the disciplines of public health education and policy. Initially, there may be focused instructional programs including e-health literacy instruction into college courses. This would enable students to locate, assess, and use reliable health information online, therefore enhancing their capacity for making wise decisions about their health. Further increasing involvement and literacy should be performed by improving access to e-health technologies and considering their user-friendliness features. e-health environments should help to develop self-management abilities.

Public health campaigns help one to have more influence over their personal health. The growing range of e-health goods emphasizes the demand of the digital health sector. Before we can encourage the use of digital technologies, we must first make certain that they are safe and secure for users’ personal information.

At long last, public health emergencies have the potential to serve as a catalyst for an increased number of individuals to cultivate their understanding of and use of e-health. Young people may be able to assist in the negotiation of digital healthcare, stimulate the acceptance of digital health, and significantly enhance public health if they are encouraged to learn about e-health and to be prepared for future emergencies.

### 4.4. Limitations and Future Research Directions

There should be certain limits acknowledged even with the efforts made in conducting and improving this research. The sample consisted of less than three hundred students; hence demographic limits could affect the generalizability of the results. Apart from that, this study centered solely on the Chinese environment and the cross-sectional nature of the research limits its power to generate causal links. Other possible elements that could be investigated in next studies for a more whole knowledge are socioeconomic level, the availability of digital infrastructure, and institutional support. Examining many people from several areas and age groups helps one to raise the generalizability of the data. To monitor e-health literacy over time, it is advised to use longitudinal studies.

While this study offers valuable insights into factors influencing e-health literacy among university students, its geographical focus on Shandong province may limit the generalizability of the findings. Shandong’s advanced digital infrastructure, educational resources, and demographic diversity likely contributed to the observed literacy levels, which may differ in regions with less developed infrastructure or distinct cultural attitudes toward technology. For example, students in remote rural areas or provinces with limited digital health resources may exhibit lower e-health literacy, potentially reducing the applicability of these findings to other regions in China.

Future research should aim to replicate this study in other provinces and regions with varying demographic and cultural characteristics. Expanding the scope of research in this way will provide a more comprehensive understanding of e-health literacy across diverse contexts and ensure the development of region-specific interventions and policies.

## 5. Conclusions

This study investigated the e-health literacy of university students, providing key insights into their proficiency levels and factors influencing their ability to navigate digital health environments. The main findings are summarized below:

- Overall Proficiency

The overall e-health literacy level among university students was moderate, with significant variability across specific skills. Students demonstrated the highest proficiency in locating relevant health information online, but challenges persisted in evaluating and utilizing the quality of digital health resources.

- Behavioral Engagement

The frequency of e-health tool usage, particularly mobile applications, increased substantially during the COVID-19 pandemic. Behavioral engagement played a pivotal role in improving e-health literacy levels.

- Key Influencing Factors

Government policies, risk perception, education, and self-management challenges were significant factors influencing e-health literacy. Demographic characteristics, such as academic qualifications and place of origin, had minimal impact, highlighting the greater importance of behavioral and resource-related factors.

- Recommendations for Interventions

Improve access to reliable e-health resources to ensure equitable availability. Integrate digital literacy training into academic curricula to enhance students’ ability to critically assess and use online health tools.

Develop policies that encourage proactive engagement with digital health technologies, addressing concerns such as data privacy and trust.

- Pandemic as a Catalyst

The COVID-19 pandemic significantly accelerated the adoption of e-health technologies, particularly among digitally proficient students, reinforcing the need for targeted interventions to build on this momentum. By addressing these findings, stakeholders can work collaboratively to enhance e-health literacy, equipping young adults with the skills and resources needed to navigate an increasingly digital healthcare landscape and make informed health decisions.

- Lessons Learned for the Future

This study underscores several critical lessons for enhancing e-health literacy in the future: 1. Public health crises can accelerate digital health adoption but must be accompanied by strategies to address barriers such as misinformation, privacy concerns, and digital inequities. 2. Integrating e-health literacy education into university curricula can empower students to critically evaluate and effectively use online health resources, fostering better health management. 3. Policy makers and educators should collaborate to develop inclusive programs that account for cultural, technological, and demographic differences, ensuring equitable access to digital health tools. 4. Future research should explore longitudinal impacts of e-health literacy and examine the role of emerging technologies, such as AI and blockchain, in enhancing digital health ecosystems.

## Figures and Tables

**Table 1:** Reliability Analysis of the e-Health Literacy Scale.

Cronbach’s Alpha Coefficient	Standardized Cronbach’s Alpha Coefficient	Item Count	Sample Size
0.928	0.931	8	249

**Table 2:** Item-Level Analysis of the e-Health Literacy Scale with Cronbach’s Alpha Coefficients After Deletion.

Concern	Average Value After Deletion of Entries	Variance After Deletion of Terms	Correlation of Deleted Items with the Total After Deletion of Items	Cronbach’s Alpha Coefficient After Deletion of Terms
I know how to find useful information about health resources on the Internet	24.811	37.331	0.631	0.924
I know how to use the internet to answer my health questions	24.663	36.257	0.746	0.92
I know what health resource information is available from the internet	24.606	36.869	0.795	0.916
I know where to find useful health resource information online	24.534	36.984	0.829	0.914
I know how to use the information I get about online health resources to help myself	24.562	36.465	0.818	0.914
I have the skills to evaluate good and bad information about online health resources	24.53	37.887	0.761	0.919
I am able to differentiate between high- and ow-quality health resource information online	24.558	37.554	0.756	0.919
I am confident in using online information for health decisions	24.606	37.836	0.736	0.92

**Table 3:** Mean Scores of e-Health Literacy Scale Items.

Concern	Mean
I know how to find useful information about health resources on the Internet	3.31 ± 1.20
I know how to use the internet to answer my health questions	3.46 ± 1.16
I know what health resource information is available from the internet	3.52 ± 1.04
I know where to find useful health resource information online	3.59 ± 0.99
I know how to use the information I get about online health resources to help myself	3.56 ± 1.06
I have the skills to evaluate good and bad information about online health resources	3.59 ± 0.98
I am able to differentiate between high- and low-quality health resource information online	3.57 ± 1.02
I am confident in using online information for health decisions	3.52 ± 1.01
Total	28.12 ± 8.46

**Table 4:** Changes in Frequency of e-Health Usage among Participants.

Frequency Changes	Subtotal	Proportion
Increased usage	151	60.64%
Reduced usage	21	8.43%
No change	77	30.92%

**Table 5:** Classification of e-Health Product Usage among Participants.

Software Classification	Subtotal	Proportion
Electronic bracelet watch	131	24%
Apps	163	29%
Mini programs (Meiyu, Ping An Good Doctor, Lilac Doctor, etc.)	130	23%
Short video platforms such as Douyin	100	18%
Other	30	5%

**Table 6:** Influencing Factors on Participants’ e-Health Usage.

Influencing Factors	Subtotal	Proportion
Risk area division	156	29%
State policy	164	31%
Public opinion orientation	123	23%
Expert advice	62	12%
Other	24	5%

**Table 7:** Degree of Conformity in Participants’ Responses.

Degree of Conformity	Subtotal	Proportion
Very inconsistent	28	11.24%
Something doesn’t match	35	14.06%
Can’t tell	50	20.08%
Some match	103	41.37%
Very consistent	33	13.25%

**Table 8:** Degree of Conformity in Participants’ Responses: “I am able to differentiate between high-and low-quality health resource information online”.

Degree of Conformity	Subtotal	Proportion
Very inconsistent	11	4.42%
Something doesn’t match	28	11.24%
Can’t tell	56	22.49%
Some match	117	46.99%
Very consistent	37	14.86%

**Table 9:** Options for the Development and Standardization of e-Health Systems. “What do you think must be addressed first in order to implement e-health-related services?”

Options	Subtotal	Proportion
It is suitable for the development and promotion of digital medical and health equipment	137	55.02%
Collection and storage of personal health information	143	57.43%
e-health records are networked nationwide	126	50.6%
Establish mechanisms to safeguard information security and privacy	120	48.19%
Standardization and standardization of medical diagnosis	103	41.37%
Strengthen supervision of relevant medical industry media	46	18.47%
Standardization of e-health-related equipment and technologies	40	16.06%

**Table 10:** Options for the Development and Standardization of e-Health Systems.

Results of the Linear Regression Analysis (*n* = 249)									
	Non-Standardized Coefficients		Standardization Factor	t	*p*	VIF	R^2^	Adjustment of R²	F
	B	Standard Error	Beta						
Constants	1.32	0.278	-	4.747	0.000 ***	-	0.192	0.168	F = 8.155 *p* = 0.000 ***
Change in frequency of use of e-health products after the outbreak	0.214	0.067	0.189	3.214	0.001 ***	1.035			
Access to e-health	0.023	0.004	0.315	5.389	0.000 ***	1.016			
Academic qualifications	−0.007	0.092	−0.005	−0.081	0.936	1.029			
Place of origin (domicile)	0.068	0.085	0.047	0.801	0.424	1.011			
Factors influencing concern about the epidemic as well as e-health	−0.02	0.008	−0.141	−2.394	0.017 **	1.031			
Difficulties encountered in self-e-health management	−0.011	0.007	−0.101	−1.724	0.086 *	1.033			
Gender	0.17	0.09	0.11	1.887	0.060 *	1.02			

Note: ***, ** and * represent 1%, 5% and 10% significance levels, respectively. Dependent variable: 4. Are you familiar with the definition of e-health

## Data Availability

Data are available upon request.
